# AN INTERSECTIONAL APPROACH TO UNDERSTANDING COMBINING WORK AND CAREGIVING ON MENTAL HEALTH

**DOI:** 10.1093/geroni/igad104.2216

**Published:** 2023-12-21

**Authors:** Samantha Brady, Taylor Patskanick, Joseph Coughlin

**Affiliations:** Massachusetts Institute of Technology, Cambridge, Massachusetts, United States; Massachusetts Institute of Technology, Cambridge, Massachusetts, United States; Massachusetts Institute of Technology, Cambridge, Massachusetts, United States

## Abstract

While an increasing number of Americans are providing unpaid care to an adult family member, the majority are balancing family care needs with employment responsibilities. Role conflict theory suggests the experience of combining family caregiving and paid work can detrimentally impact mental health, while role enhancement theory suggests that occupying both social roles can alleviate the burden of caregiving. Prior work finds support for both theories, however, there is currently a lack of causal evidence examining how mental health is affected by combining caregiving and employment roles. Additionally, while gendered differences and differences across racial identities in the family caregiving experience are well-documented, even fewer studies have taken an intersectional approach in understanding shifts in mental health when caregiving while employed. The current study uses a causal methodology to examine how the mental health of working adults is impacted when they transition into a family caregiving role and how these impacts vary at the intersections of racial and gender identity. Data are from the Health and Retirement Study (2004-2018), a nationally representative survey of U.S. adults aged 50+. Results from fixed effects models support role conflict theory, suggesting when working adults begin family caregiving they experience an increase in depressive symptoms. Additional analysis using an intersectional lens finds this result applied only to White working women who transitioned into a parental caregiving role. Combining care with paid work did not appear to impact depressive symptoms for employed Black women, Black men, and White men when they began caregiving.

